# *Dicer*, a new regulator of pluripotency exit and LINE‐1 elements in mouse embryonic stem cells

**DOI:** 10.1002/2211-5463.12174

**Published:** 2017-01-11

**Authors:** Maxime Bodak, Daniel Cirera‐Salinas, Jian Yu, Richard P. Ngondo, Constance Ciaudo

**Affiliations:** ^1^ Department of BiologyRNAi and Genome IntegrityIMHSSwiss Federal Institute of Technology ZurichZurichSwitzerland; ^2^ Life Science Zurich Graduate SchoolMolecular Life Science ProgramUniversity of ZurichSwitzerland; ^3^ Life Science Zurich Graduate SchoolMolecular and Translational Biomedicine ProgramUniversity of ZurichSwitzerland

**Keywords:** *Dicer*, LINE‐1 retrotransposition, mouse embryonic stem cells, transposable elements

## Abstract

A gene regulation network orchestrates processes ensuring the maintenance of cellular identity and genome integrity. Small RNAs generated by the RNAse III DICER have emerged as central players in this network. Moreover, deletion of *Dicer* in mice leads to early embryonic lethality. To better understand the underlying mechanisms leading to this phenotype, we generated *Dicer*‐deficient mouse embryonic stem cells (mESCs). Their detailed characterization revealed an impaired differentiation potential, and incapacity to exit from the pluripotency state. We also observed a strong accumulation of LINE‐1 (L1s) transcripts, which was translated at protein level and led to an increased L1s retrotransposition. Our findings reveal *Dicer* as a new essential player that sustains mESCs self‐renewal and genome integrity by controlling L1s regulation.

**Abbreviations**
AP: alkaline phosphatase
EBs: embryoid bodies
KO: knockout
LIF: leukemia inhibitory factor
LINE‐1, L1s: long interspersed nuclear element 1
LTR: long terminal repeat
mESCs: mouse embryonic stem cells
miRNA: microRNA
OSN: *Oct4‐Sox2‐Nanog*
Pre‐miRNA: precursor miRNA
RNAi: RNA interference
SINE: short interspersed nuclear element
siRNA: small‐interfering RNA
TE: transposable element
WT: wild‐type

Since its discovery in 2001 1, extensive studies revealed DICER as a key player of RNA interference (RNAi) processes. Indeed, this RNase III protein is essential for microRNAs (miRNAs) and small‐interfering RNAs (siRNAs) biogenesis 2, 3, 4. These eukaryotic small RNAs are central players in many biological processes by mediating gene silencing at transcriptional or post‐transcriptional levels 5. They are also essential actors of early mammalian development as key regulators of cell cycle and proliferation 6. Moreover, particular miRNAs are also involved in embryonic stem cell fate regulation by promoting self‐renewal and differentiation 7, 8, 9, 10, 11. The disruption of the *Dicer* gene leads to early embryonic lethality at the implantation stage, emphasizing its critical role during mouse early development 12, 13. Besides, RNAi pathways can act as defense mechanisms against endogenous and exogenous factors like transposable elements (TEs) and viruses 14, 15, 16. In mammals, first evidence of TEs regulation by RNAi was reported in *Dicer‐*depleted preimplantation mouse embryos, where specific subclasses of TEs were up‐regulated 14. Nevertheless, the exact mechanisms by which RNAi players could act on TEs and the consequences of this regulation during early development remain unclear.

To better understand the functions of *Dicer* during early mammalian development, we used mouse embryonic stem cells (mESCs) as a model system. Derived from the inner cell mass of mouse blastocyst, mESCs present two substantial advantages: first, they can be maintained in a pluripotent state or conversely be differentiated into the three germ layers depending on the culture conditions 17. Thus, making them a suitable model to study mouse embryonic developmental stages *in vitro*, otherwise difficult to assess *in vivo*. Second, TEs are not submitted to their major regulatory mechanisms at the blastocyst stage. Both, the DNA methylation and the PIWI‐interacting RNA (piRNA) silencing taking place in somatic and germ cells, respectively, are absent at this stage 18, 19—suggesting the existence of alternative regulatory pathways. Therefore, mESCs represent a relevant model to study TEs regulation during mouse early development as well.

Long INterspersed Element‐1 (LINE‐1 or L1s), long terminal repeat (LTR), and short interspersed nuclear element (SINE) are the three main subgroups composing the retrotransposons family, which are the major class of TEs represented in mammalian genomes 20, 21, 22, 23. L1s are the most abundant TEs in human and mouse genomes (21% and 17%, respectively) 21, 22. They belong to the autonomous retrotransposon category, as they code for the machinery necessary for the RNA intermediate production, its reverse transcription, and integration into a new genomic location 24. Although the large majority of L1s are inactive 25, it is estimated that around 3000 full‐length L1s have maintained their ability to retrotranspose in the mouse genome 26, 27, 28. Active full‐length L1s, via their retrotransposition ability, can act as mutagens by inserting into exons, or induce aberrant splicing or exon skipping by inserting into introns 29. Therefore, they can deeply influence the genome, in beneficial and detrimental ways 30, and need to be tightly controlled.

In order to investigate the roles of DICER during mouse early development, we generated new *Dicer* knockout mESCs mimicking previously described *Dicer* ^*Cre‐loxP*^ mutants 31, 32. Their detailed characterization highlighted their inability to differentiate and revealed for the first time their incapacity to exit from the pluripotent state and a factual reinforcement of their pluripotency network. Additionally, transcriptome analysis of wild‐type (WT) and *Dicer*_KO mESCs unveiled an up‐regulation of LINE‐1 transcripts. This increase of L1s mRNAs was translated at the protein level and led to an augmentation of their retrotransposition rate. Taken together, our experiments highlight critical roles of *Dicer* in the regulation of the pluripotency network and the control of LINE‐1 elements in mESCs.

## Materials and methods

### Culture and *in vitro* differentiation of mESCs

E14TG2a (ATCC CRL‐1821) line has been used as WT mESCs. Cell culture and embryoid body (EB) differentiation assays were performed as described in 33. Unless otherwise specified, mESCs were routinely cultured in serum + LIF condition.

### Generation of *Dicer*_KO mESCs using CRISPR/Cas9

*Dicer*_KO mESCs were generated from E14TG2a mESCs using a paired CRISPR/Cas9 strategy as described in 34. Specific CRISPR/Cas9 sgRNAs have been generated using the e‐crispr software 35 or chosen from an established library 36 and cloned into the plasmid pX330‐U6‐Chimeric_BB‐CBh‐hSpCas9 37 using the *Bbs*I restriction site. mESCs were single cell sorted 48 h after transfection. All the primers used for the CRISPR/Cas9 are described in Table S1. All newly generated plasmids are described in Table S2. All designs are based on the latest mouse genome assembly (GRCm38/mm10) provided by the UCSC Genome browser http://genome.ucsc.edu/.

### Genomic DNA extraction and PCR

Genomic DNA was extracted from 1.10^6^ mESCs using Roti^®^ Phenol/Choloroform/Isoamyl Alcohol. Each PCR reaction has been performed using 50 ng of genomic DNA. Genotyping PCR primers sequences are described in Table S1.

### RT‐qPCR analysis

RT‐qPCR analysis was performed as described in 33. All the primers used for the RT‐qPCR assays are described in Table S1.

### Immunoblotting analysis and antibodies

Immunoblotting analysis was performed as described in 33. All the antibodies used for the immunoblot assays are described in Table S3. In the case of subsequent reprobing, polyvinylidene difluoride membranes were reactivated into methanol, and then stripped with successive 0.2 m NaOH washes. Finally, membranes were blocked during 1 h at room temperature using a 5% milk solution, before reprobing with a second primary antibody.

### Low molecular weight northern analysis

Low molecular weight northern analysis were performed as described in 38 using 10 μg of total RNA extracted from 1.10^6^ mESCs pellets using TRizol^®^ Reagent. Membranes were EDC cross‐linked. For subsequent reprobing, membranes were stripped with boiling 0.1% SDS. All the DNA oligonucleotides complementary to miRNAs and U6 small RNA, used for the probes generation, are listed in Table S1.

### High molecular weight northern analysis

Total cellular RNA was extracted from 1.10^6^ mESCs pellets using TRizol^®^ Reagent. About 30 μg of total RNA were resolved on a denaturing 1% agarose gel with 1% formaldehyde, and capillary transferred overnight on a positively charged nylon membrane using 20X saline sodium citrate solution (SSC). Membrane was cross‐linked by UV radiation. Prehybridizations and hybridizations were both performed in PerfectHyb™ Plus Hyridization Buffer at 42°C. All washes were performed in SSC 2X, SDS 0.1%. The radiolabeled L1_probe for the detection of full‐length L1 transcripts was produced by random‐priming of a PCR product generated from E14TG2a mESCs genomic DNA using specific primers 39 described in Table S1.

### RNA sequencing

Total cellular RNA was extracted from 1.10^6^ mESCs pellets using TRizol^®^ Reagent. The quality of isolated RNA was determined with a Bioanalyzer 2100 (Agilent, Santa Clara, CA, USA). Up to 2 μg of polyA purified RNA was used for the library preparation, done with the TruSeq Paired‐end stranded RNA Preparation Kit (Illumina, San Diego, CA, USA). The library preparation and sequencing (Illumina HiSeq 2000) were performed at the Functional Genomics Center Zurich (FGCZ). Paired end sequencing generated about 2 × 60 millions of reads per library. Reads from RNA sequencing were first preprocessed by trimmomatic (v0.32) 40 to remove low‐quality ends and adapters using default settings. Reads were aligned to the mouse genome mm10 by STAR (v2.4.2a) 41 allowing for two mismatches and up to 3000 multiple‐hits. FeatureCounts (v1.4.5‐p1) 42 was used to count reads for genes (Ensembl GRCm38.78), ignoring reads on overlapping region and the plot was generated using ggplot2 (v1.0.1) 43. tetoolkit (v1.5) 44 was used to count reads for repeat elements, accounting for multiple‐hit reads and RPKM were calculated by using edgeR 45. Complete RNA sequencing data of WT and *Dicer_*KO mESCs are available on the NCBI GEO database (GEO: GSE78971 for WT and GEO: GSE78973 for *Dicer*_KO).

### Proliferation assay

Cells were plated in 96‐well plate at a density of 15 000 cell·cm^−2^ and proliferation was assessed every day during 4 days using the CellTiter‐Glo^®^ Luminescent Cell Viability Assay.

### Cell cycle analysis

Cell cycle analysis was performed as described in 46.

### Apoptotic cell population analysis

Apoptotic cell population analysis was performed as described in 47 (direct DNA staining in PI hypotonic solution and subsequent analysis by FACS).

### Exit from pluripotency assay

Cells were plated in six‐well plate at a density of 4500 cells·cm^−2^ and cultured in 2i medium (N_2_B_27_ (Cellartis) complemented with 50 U·mL^−1^ of penicillin and 0.05 mg·mL^−1^ of streptomycin) containing or not the following inhibitors cocktail: PD032591 at 1 μm final concentration, CHIR99021 at 3 μm final concentration, and 1000 U·mL^−1^ of leukemia inhibitory factor (LIF). The alkaline phosphatase (AP) staining was performed using the Leukocyte Alkaline Phosphatase kit (Sigma, St. Louis, MO, USA). For the clonal AP quantification, entire six‐well plates used for AP staining assays were first scanned to capture the total plate area in a single image. Images were then processed using the ImageJ software 48. The number of AP‐positive colonies was calculated on threshold intensity (default parameters) of inverted regions that were user‐selected (full well – identical areas between conditions) using the Analyze Particles tools (default parameters).

### Immunostaining

Cells were washed once with PBS1X, incubated 10 min at 37°C with 4% paraformaldehyde solution for fixation and then incubated 15 min on ice in a 90% methanol solution for permeabilization. Next, cells were incubated 1 h at room temperature with the primary and secondary antibody, successively. Between incubation steps, cells were washed once with PBS1X. Antibodies used for the immunostaining assays are described in Table S3. Cells were analyzed by flow cytometry using selective gating to exclude the doublets of cells.

### Retrotransposition assay

Cells were plated at a density of 20 000 cells·cm^−2^ per well 24 h before transfection with 0.5 μg of plasmid DNA using Lipofectamine^®^ 2000 reagent according to the manufacturer's instructions. Antibiotic selection started 24 h after transfection using puromycin‐containing medium (1 μg·μL^−1^) and maintained during the entire assay. Every week, cells were trypsinized and replated at a density of 5500 cells·cm^−2^ into a new gelatin‐coated six‐well tissue culture plate and the remaining cells were used for subsequent FACS analysis. In total, WT cells have been passaged six times and *Dicer_*KO mESCs four times (due to their proliferation defects). Cells were analyzed by FACS using selective gating excluding doublets of cells (Fig. 5E). The gating for EGFP‐positive and EGFP‐negative cells was determined by analyzing cells transfected with: a plasmid coding EGFP (positive control) and a puromycin‐resistance gene and a plasmid coding only a puromycin‐resistance gene (negative control), respectively (Fig. 5B). A final gate of 3.10^4^ events per sample was acquired.

## Results

### Generation and validation of *Dicer*_KO mESCs

**Figure 1:**
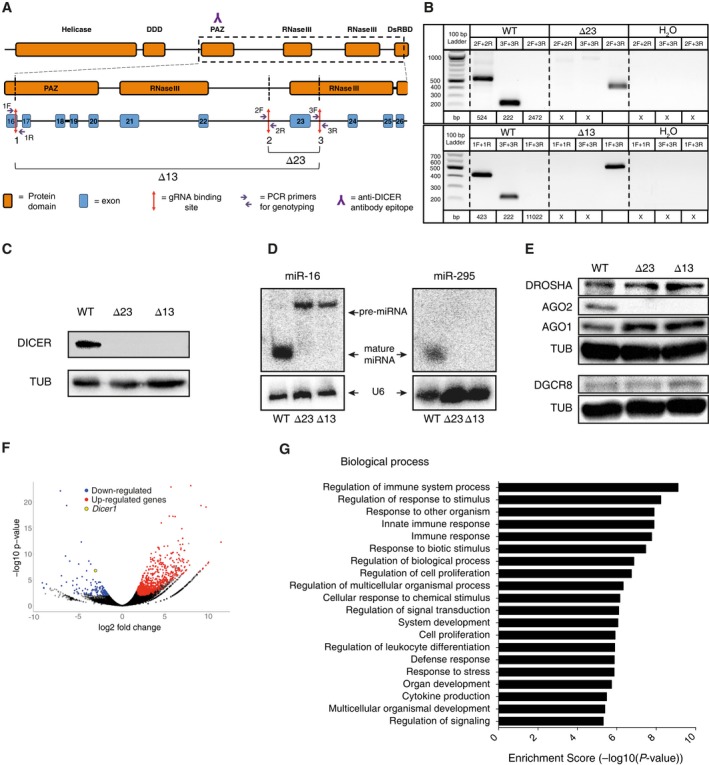
Generation of *Dicer*_KO mESCs using the paired CRISPR/Cas9 approach. (A) CRISPR/Cas9 design. The structure of the DICER protein is shown at the top, with the genomic regions corresponding to area from the PAZ domain to the second RNAse III domain below. The anti‐DICER antibody recognizes the 961–975 amino acids region of the PAZ domain. Three CRISPR/Cas9 single guide RNAs (sgRNAs), targeting the *Dicer* gene were designed: sgRNA 1 in the exon 16, sgRNA 2 in‐between exon 22 and 23, and sgRNA 3 in‐between exon 23 and 24. The combination of the sgRNAs 1 and 3 deleted the region between the PAZ domain and the second RNAse III domain (Δ13), and the sgRNAs 2 and 3 erased the second catalytic RNase III domain (Δ23). Specific genotyping primers have been designed around each sgRNA‐binding sites allowing a PCR screening of positive candidates for the deletions, used in (B). (B) PCR on genomic DNA of WT and *Dicer*_KO mESCs. Deletions Δ23 and Δ13 were confirmed by the presence of DNA amplicons of 413 bp and 492 bp, respectively. (C) Immunoblot analysis of DICER protein levels in WT and *Dicer*_KO mESCs. For protein normalization, α‐Tubulin (TUB) was used as a loading control. Representative blot of three independent experiments is shown. (D) Northern blot analysis using WT and *Dicer*_KO mESCs total RNA extract probed with specific miR‐295 and miR‐16 probes. Pre‐miRNA and mature miRNAs are indicated by arrows. Samples were probed with a U6‐specific probe as loading control. Representative blot of three independent experiments is shown. (E) Immunoblot analysis of DICER, DROSHA, DGCR8, AGO2, and AGO1 protein levels in WT and *Dicer*_KO mESCs. For protein normalization, α‐Tubulin (TUB) was used as a loading control. Representative blot of three independent experiments is shown. (F) Volcano plot showing the global transcriptional changes in *Dicer*_KO vs WT mESCs. Each circle represents one gene. The *x*‐axis shows the log fold change and the *y*‐axis shows the log10 of the *P*‐value. Differentially expressed genes are represented by colored circles and are defined by a fold change superior to 2 and a false discovery rate inferior to 0.01. (G) Graphical demonstration of associated biological processes of differentially expressed genes in *Dicer*_KO relative to WT mESC samples. The *y*‐axis displays the biological process categories that are identified in the analysis. The *x*‐axis shows the enrichment score, which is the value of −log10(*P*‐value). Functions are listed from the most enriched to least. The top 20 biological process categories are displayed. Pathways analysis has been performed using the Consensus PathDB‐mouse database (CPDB) 93, 94.

We first generated two independent *Dicer1* knockout (*Dicer*_KO) mESC lines using the CRISPR/Cas9 technology 37, 49, 50. We opted for the paired CRISPR/Cas9 approach 34, 51 and generated two independent genomic deletion events Δ23 and Δ13 (Fig. 1A). Independent mESC clones were isolated and genomic deletions were confirmed by PCR (Fig. 1B) 34. Immunobloting analysis validated the absence of DICER protein in both mutant mESC lines (Fig. 1C). The nonfunctionality of the *Dicer* knockouts was confirmed with the absence of two endogenous mature miRNAs: *miR‐16* and *miR‐295* (Fig. 1D) 38, 52, 53. The accumulation of *miR‐16* precursors (pre‐miRNA) in both *Dicer*_KO mESCs proved the functionality of the microprocessor complex (DROSHA and DGCR8; Fig. 1D). Furthermore, immunoblotting revealed no differences in the expression of the other RNAi pathway proteins: DROSHA, DGCR8, and AGO1, between *Dicer*_KO mutants and WT mESCs (Fig. 1E) 2. However, we observed dramatically reduced AGO2 levels in both *Dicer*_KO mutants, consistent with the lack of mature miRNAs leading to the destabilization of the AGO2 protein 54. Finally, the analysis of the RNA sequencing data confirmed the loss of *Dicer* mRNA in both *Dicer_KO* mutants and profound changes in the transcriptome with 879 genes differentially expressed (Fig. 1F and Table S4), involved in many biological pathways (Fig. 1G). Most of the genes differentially expressed were as expected up‐regulated (80%), due to the essential role of DICER in post‐transcriptional gene silencing mechanisms mediated by miRNAs (Fig. 1F and Table S4). Taken together, these experiments validate the successful generation of two new independent *Dicer* knockout lines.

**Figure 2:**
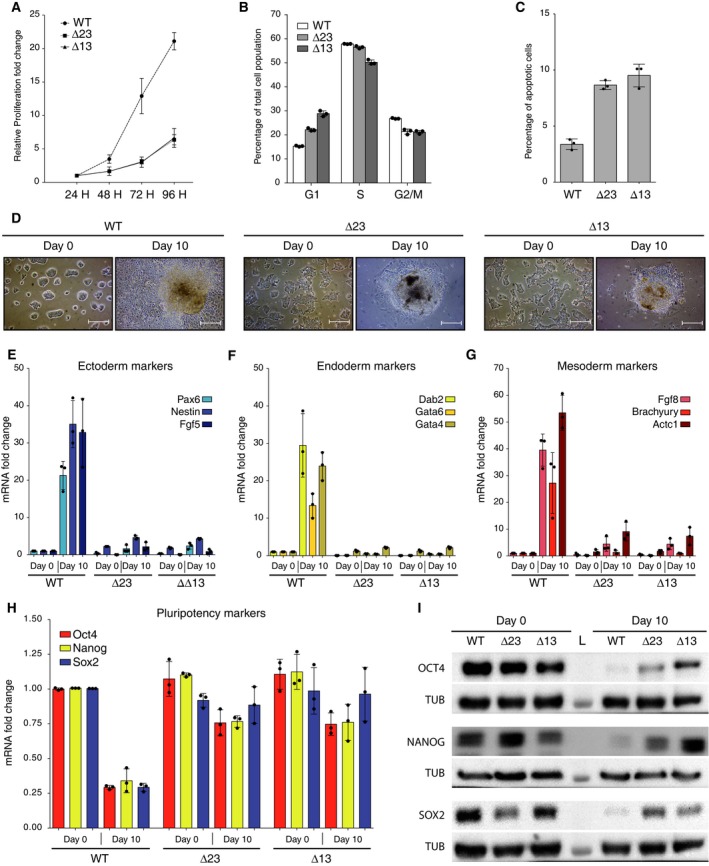
Characterization of newly generated *Dicer*_KO mESCs. (A) Proliferation assay of WT and *Dicer*_KO mESCs. For each cell line, data are shown as the fold change in the number of metabolically active cells compared to the first measurement done 24 h after the plating. Data are represented as mean ± SD (*n* = 3). (B) Cell cycle analysis of WT and *Dicer*_KO mESCs. Data are represented as mean ± SD (*n* = 3). (C) Apoptotic cell population analysis of WT and *Dicer*_KO mESCs. Data are represented as mean ± SD (*n* = 3). (D) Visualization of WT and *Dicer*_KO mESCs at Day 0 (upper panel) and at Day 10 (lower panel) of embryoid body (EB) differentiation. Scale bar = 50 μm. (E) RT‐qPCR analysis of three ectoderm markers: *Pax6*,* Nestin*, and *Fgf5* mRNAs in WT and *Dicer*_KO mESCs. The data are shown as the fold change compared to WT cells after normalization to the *Gapdh* housekeeping gene at Day 0. Data are represented as mean ± SD (*n* = 3). (F) RT‐qPCR analysis of three endoderm markers: *Dab2*,* Gata6* and *Gata4* mRNAs in WT and *Dicer*_KO mESCs. The data are shown as the fold change compared to WT cells after normalization to the *Gapdh* housekeeping gene at Day 0. Data are represented as mean ± SD (*n* = 3). (G) RT‐qPCR analysis of three ectoderm markers: *Fgf8*,* Brachyury*, and *Actc1* mRNAs in WT and *Dicer*_KO mESCs. The data are shown as the fold change compared to WT cells after normalization to the *Gapdh* housekeeping gene at Day 0. Data are represented as mean ± SD (*n* = 3). (H) RT‐qPCR analysis of pluripotency markers: *Oct4 (Pou5f1)*,* Nanog*, and *Sox2* mRNAs in WT and *Dicer*_KO mESCs before and after 10 days of EB differentiation. The data are shown as the fold change compared to WT cells after normalization to the *Gapdh* housekeeping gene at Day 0. Data are represented as mean ± SD (*n* = 3). (I) Immunoblot analysis of OCT4, NANOG, and SOX2 protein levels in WT and *Dicer*_KO mESCs at Day 0 and Day 10 of EB differentiation. For protein normalization, α‐Tubulin (TUB) was used as a loading control. L = Protein Ladder. Representative blot of three independent experiments is shown.

Next, we characterized our *Dicer*_KO mESCs and evaluated their proliferation rate. After 3 days, both *Dicer* mutants showed a strongly impaired proliferation (twofold) compared to WT mESCs. The proliferation defect was exacerbated after 4 days (threefold), confirming the delay (Fig. 2A). The cell cycle distribution analysis revealed an accumulation in G1‐phase in both *Dicer* mutants, suggesting an impaired G1/S transition as the direct cause of the proliferation defect (Fig. 2B). Indeed, many miRNAs regulate the entry and G1–S‐phase transition 55, making this observation consistent with the lack of miRNAs of *Dicer*_KO mESCs. Interestingly, *Dicer*_KO mESCs also showed a twofold increase of the apoptotic cells population compared to WT (Fig. 2C). Importantly, it has been also documented that numerous miRNAs are involved in apoptosis regulation 56. In conclusion, newly generated *Dicer*_KO mESCs proliferate much slower due to a G1‐phase arrest and an increased apoptosis rate.

Previously characterized *Dicer* ^*Cre‐loxP*^ mutant mESCs failed to contribute to the embryo development when injected into WT blastocyst and could not differentiate *in vitro* 31. To understand the molecular mechanisms leading to this differentiation defect, we first tested the ability of our mutants to form EBs *in vitro*. When cultured in suspension in the absence of cytokine LIF, mESCs form cell aggregates known as EBs, differentiating toward the three germ layers 17. After 10 days of EBs differentiation (Day 10), WT mESCs produced fully developed EBs, while *Dicer*_KO mutants formed cells aggregates without morphological evidence of differentiation (Fig. 2D). RT‐qPCR analysis performed at Day 0 and Day 10 with specific primers for the differentiation markers, *Pax6*,* Nestin*,* Fgf5* (ectoderm; Fig. 2E); *Dab2*,* Gata6*,* Gata4* (endoderm; Fig. 2F); and *Fgf8*,* Brachyury*,* Actc1* (mesoderm; Fig. 2G), revealed that *Dicer*_KO mutants failed to differentiate to any of the three germ layers (Fig. 2E–G). Additionally, we assessed the expression of the pluripotency markers, *Oct4 (Pou5f1)*,* Sox2*, and *Nanog* (OSN), at the mRNA and protein levels. These transcription factors constitute the core of the stem cell pluripotency network and are strongly expressed in undifferentiated mESCs and silenced during the differentiation process 57, 58. RT‐qPCR analysis revealed a strong decrease of OSN mRNAs in WT mESCs after 10 days of differentiation (Fig. 2H). However, *Dicer*_KO mESCs presented an abundant accumulation of those mRNAs even after 10 days of differentiation (Fig. 2H). Immunoblotting analysis showed similar protein levels of these transcription factors in *Dicer* mutants and WT mESCs at Day 0 (Fig. 2I). More importantly, OCT4, NANOG, and SOX2 proteins were still expressed at Day 10 in both *Dicer* mutants, whereas no or very weak signals were observed in WT mESCs (Fig. 2I). These results confirm that *Dicer* is indeed necessary for the differentiation of mESCs.

### *Dicer* is essential to exit the pluripotent state of mESCs

**Figure 3:**
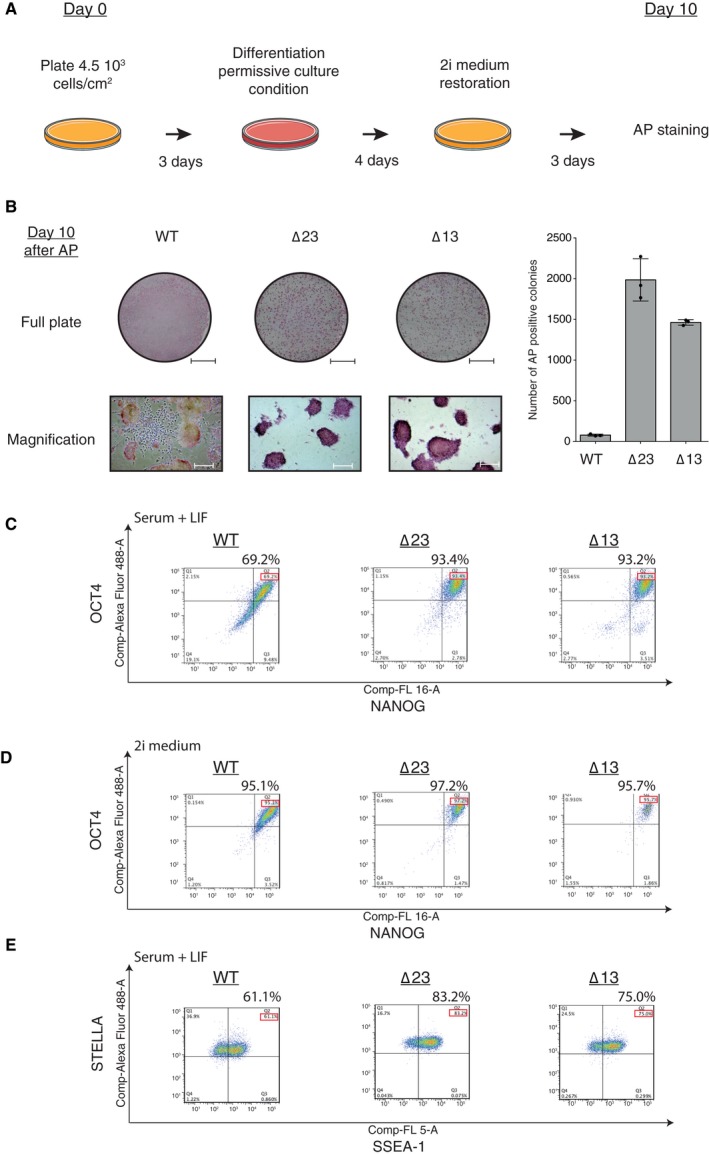
*Dicer* is essential for mESCs to exit from the pluripotent state. (A) Schematic design of the exit from pluripotency experiment. (B) Left panel corresponds to the visualization of WT and *Dicer*_KO mESCs after the alkaline phosphatase (AP) staining: full six‐well plate (scale bar = 1 cm) and magnified (scale bar = 50 μm). Representative pictures of three independent experiments are shown. Right panel displays the clonal AP quantification from whole well pictures from three independent exit from pluripotency assays. The data are shown as the number of AP positives colonies counted. Data are represented as mean ± SD (*n* = 3). (C) Flow cytometry analysis of pluripotent factors OCT4 and NANOG coexpression in WT and *Dicer*_KO mESCs in serum + LIF condition. Representative analysis of three independent experiments. (D) Flow cytometry analysis of transcription factors OCT4 and NANOG in WT and *Dicer*_KO mESCs in 2i condition. Representative analysis of three independent experiments. (E) Flow cytometry analysis of pluripotent factors STELLA and SSEA‐1 coexpression in WT and *Dicer*_KO mESCs in serum + LIF condition. Representative analysis of three independent experiments.

For their commitment to differentiation, mESCs have to exit self‐renewal state, repress the pluripotency network and initiate specific cellular lineage programs 59. The high expression of the pluripotency core proteins observed at Day10 of EB differentiation (Fig. 2H) pointed toward a failure of our mutants to suppress the pluripotency network and to exit the pluripotent state. To test this hypothesis, we performed an exit from pluripotency assay 60, 61, 62. Both *Dicer*_KO and WT mESCs were cultured during 3 days in a chemically defined medium (2i medium), containing selective GSK3β and MEK 1/2 inhibitors and LIF, to enhance viability of mESCs and to increase maintenance of pluripotency 63. Subsequently, the cells were cultured for 4 days in a differentiation‐permissive medium (2i medium without inhibitors and LIF) and afterwards, the 2i medium was restored for three more days before AP staining was performed (Fig. 3A). Only pluripotent stem cells can survive and express AP in 2i medium. To account for the strong proliferation defect of the *Dicer*_KO lines, we extended the permissive culture of the original protocol 62 from 3 to 4 days. After the exit from pluripotency assay, WT mESCs did not form colonies resembling embryonic stem cells and were AP negative, indicating that these cells committed to differentiation properly (Fig. 3B). In contrast, both *Dicer* mutants formed distinct AP‐positive colonies (Fig. 3B), demonstrating for the first time that *Dicer*_KO mESCs were still able to proliferate in 2i medium after 4 days in permissive medium. The quantification of the total cell population revealed a strong increase (20‐fold) of AP‐positive colonies for both *Dicer* mutants compared to WT mESCs (Fig. 3B). This result indicates that *Dicer*_KO mESCs retained their self‐renewal potential and remained undifferentiated in permissive conditions. Therefore, *Dice*r_KO mESCs failed to exit from the pluripotent state or presented a strong delay for their commitment.

In order to investigate the stemness status of our mutants, we assessed the expression of pluripotency and stem cell factors in different culture conditions 64, 65. OCT4/NANOG coimmunostaining flow cytometry analysis revealed that *Dicer*_KO mESCs cultured in serum + LIF condition presented a significant enrichment of cells coexpressing the pluripotent factors compared to WT mESCs (Fig. 3C). Furthermore, *Dicer*_KO mESCs presented similar coexpression levels when cultured in serum + LIF or 2i condition (Fig. 3D), thus indicating a reinforced pluripotency network compared to WT mESCs 65, 66. Additionally, similar enrichments were observed for the coexpression of two other pluripotent markers STELLA and SSEA‐1 (Fig. 3E) 67, 68. Altogether, these observations reveal that *Dicer* depletion leads to a strengthening of the pluripotency network.

### LINE‐1 elements are strongly up‐regulated in *Dicer*_KO mESCs

**Figure 4:**
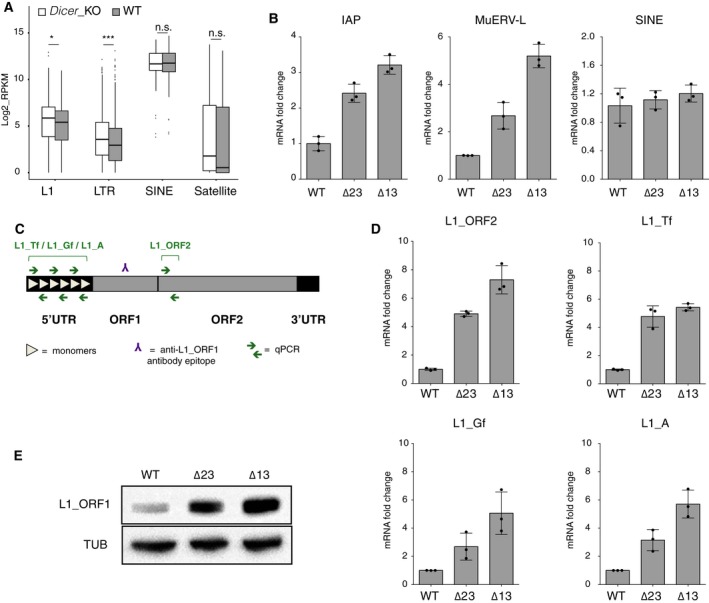
LINE‐1 elements are strongly up‐regulated in *Dicer*_KO mESCs. (A) Boxplot representing the log2 of Reads Per Kilobase per Million (RPKM) of the three major retrotransposon subclasses in WT and *Dicer*_KO mESCs. As a control, we used satellite repeats, which do not belong to the transposable element (TE) family. Statistical analysis has been performed using a two‐tailed *t*‐test. n.s., not significant, *P‐value < 0.05, ***P‐value < 0.005. (B) RT‐qPCR analysis of two LTR types IAP, MuERV‐L and SINE mRNAs in WT and *Dicer*_KO mESCs. The data are shown as the fold change compared to WT cells after normalization to the *Gapdh* housekeeping gene. Data are represented as mean ± SD (*n* = 3). (C) Schematic representation of a murine L1. A full active element is about 7 kb in length and composed of a 5′UTR, two ORFs, and a 3′UTR. In mice, three active L1s subfamilies can be distinguished: Tf, Gf and A 26, 27, 95, which are defined by the variable sequence and numbers of monomers (tandem repeat units of 200 bp) contained in their 5′UTR 96. RT‐qPCR primers for overall L1s expression assessment have been designed in ORF2, and specific RT‐qPCR primers for each L1s subfamily have been designed in the 5′UTR, used in (D). (D) RT‐qPCR analysis of overall L1s and specific L1 subfamily mRNAs in WT and *Dicer*_KO mESCs. The data are shown as the fold change compared to WT cells after normalization to the *Gapdh* housekeeping gene. Data are represented as mean ± SD (*n* = 3). (E) Immunoblot analysis of L1_ORF1 protein levels in WT and *Dicer*_KO mESCs. For protein normalization, α‐Tubulin (TUB) was used as a loading control. Representative blot of three independent experiments are shown.

Interestingly, the analysis of the *Dicer* mutant transcriptomes revealed a significant accumulation of two particular TEs subclasses transcripts: L1s and LTR, compared to their WT counterparts (Fig. 4A). These observations are consistent with earlier reports showing the accumulation of transcripts from these two specific retrotransposon subgroups after *Dicer* knockout or knockdown during mouse early development 14, 31. However, we observed no difference in the expression of the SINE subclass (Fig. 4A). These observations were confirmed in our *Dicer* mutants by RT‐qPCR (Fig. 4B). We focused our interest on the L1s subclass because they are the most abundant TEs in the mouse genome, and decided to monitor L1s in our system at mRNA and protein levels (Fig. 4C–E) 33. RT‐qPCR performed with primers designed in the ORF2 (L1_ORF2; Fig. 4C and Table S1) showed an eightfold increase of L1s mRNA accumulation in both *Dicer* mutant compared to WT mESCs (Fig. 4D). Using qPCR primers specific for each L1s subfamily (L1_Tf, L1_Gf and L1_A; Fig. 4C and Table S1), we were able to observe an accumulation of all L1s subtypes in *Dicer* mutant mESCs compare to WT cells (sixfold for the L1_Tf subfamily, fourfold for both, L1_Gf and L1_A subfamilies; Fig. 4D). Additionally, L1_ORF1 protein (derived from active murine L1s) was also strongly up‐regulated in *Dicer* mutant mESCs (Fig. 4E). We concluded that in the absence of DICER, all L1s subclasses are up‐regulated at mRNA and protein levels.

### DICER restricts LINE‐1 retrotransposition in mESCs

**Figure 5:**
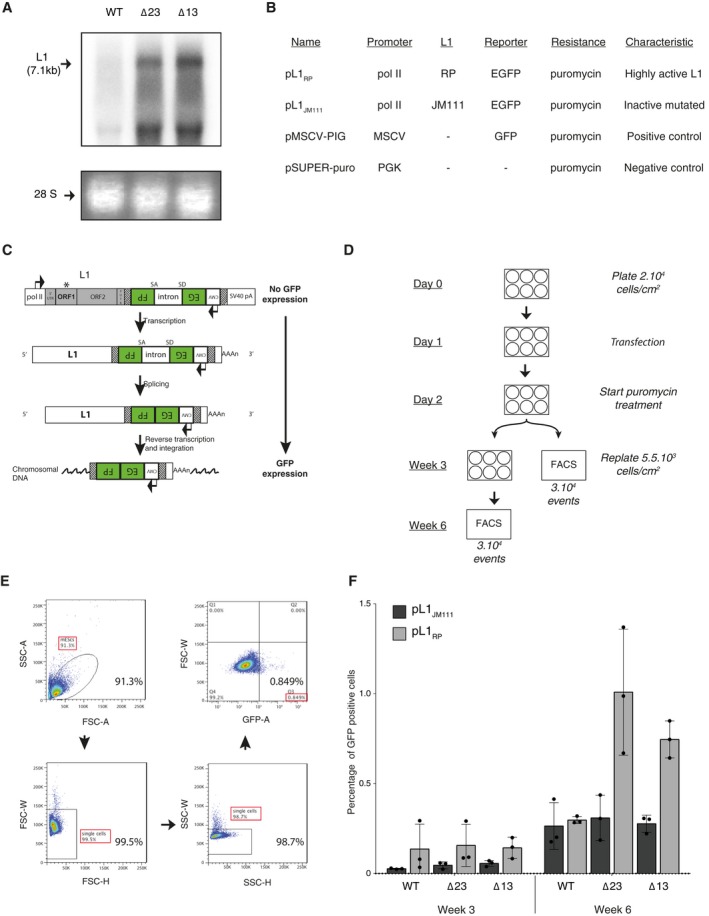
DICER restricts LINE‐1 retrotransposition in mESCs. (A) Northern blot analysis using WT and *Dicer*_KO mESCs total RNA extract probed with a specific L1_probe. Full‐length L1s transcripts are indicated with an arrow. Ethidium bromide staining before transfer was used to confirm equal loading. 28S RNA is shown as a loading control. (B) Description of the different plasmids used for the L1 EGFP‐based retrotransposition assay. (C) Schematic representation of the L1 EGFP‐transgene and its retrotransposition (adapted from 73). The L1‐EGFP transgene (pL1_RP_) consists of a human L1_RP_ element driven by the mouse RNA pol II promoter in addition to its endogenous 5′UTR. This element is coupled to an EGFP gene directed in the antisense orientation and interrupted by the mouse γ‐globin intron in the same transcriptional orientation as the L1. Therefore, when the L1‐EGFP transgene transcript is processed, the mouse γ‐globin intron is spliced out and the EGFP gene can be expressed after reverse transcription and integration into the genomic DNA. In the case of retrotransposition events, mESCs will express EGFP. In the negative control (pL1_JM__111_), the L1_RP_ element has been replaced by the L1_JM__111_ element. The L1_JM__111_ element is a nonfunctional L1 transgene consisting in a human L1 mutated in ORF1 (*) 70, abrogating its retrotransposition activity. (D) Retrotransposition assay experiment design and time line in mESCs. (E) Flow cytometry gating strategy for the analysis of GFP‐positive cells in (F). We first selected the mESC population and subsequently excluded the doublets in both dimensions. The data from the first triplicate of Δ13 mESCs transfected with the pL1_RP_ (week 6) plasmid were used to represent the gating strategy. The gating for EGFP‐positive and EGFP‐negative cells was determined by analyzing cells transfected with: a plasmid coding EGFP and a puromycin‐resistance gene and a plasmid coding only a puromycin‐resistance gene respectively described in (B). 3.10^4^ events per samples were set as a final gate. (F) Histograms summarizing the FACS analysis of the retrotransposition of pL1_RP_ and the pL1_JM__111_ transgenes in WT and *Dicer*_KO mESCs at week 3 and week 6 after transfection. The data are shown as percentage of GFP‐positive cells. Data are represented as mean ± SD (*n* = 3).

**Figure 6:**
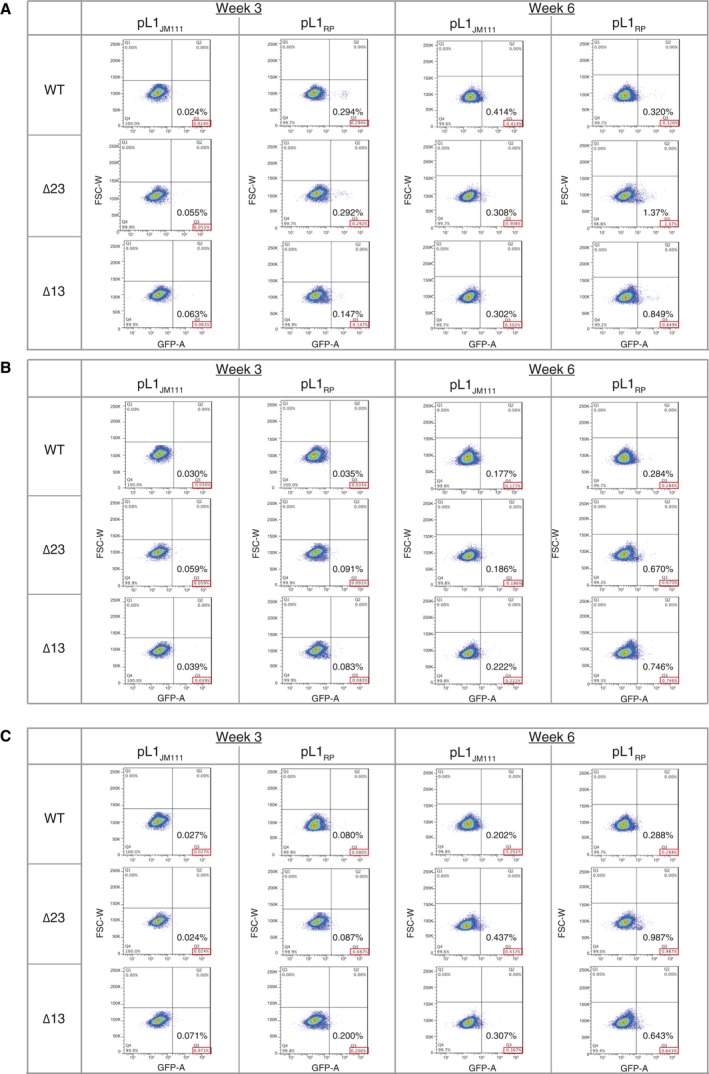
Original plots from the L1 retrotransposition assay FACS analysis. GFP‐positive cells gating strategy used for the FACS analysis of the plots generated during the L1 retrotransposition assay (FSC‐W vs GFP‐A). Cells were first gated for living population (SSC‐A vs FSC‐A) and then gated for single events (FSC‐H vs FSC‐W) and (SSC‐H vs SSC‐W). Plots for each experiment are shown in A, B, and C. L1 retrotransposition assay was performed in triplicate. (A) First triplicate. (B) Second triplicate. (C) Third triplicate.

To investigate if the increased expression of L1s could result in an augmentation of their retrotransposition rate, we first performed high molecular weight northern blotting to monitor full‐length L1s transcripts, which constitute retrotransposition‐competent intermediates. We observed a strong accumulation of L1s full‐length transcripts in *Dicer*_KO mESCs (Fig. 5A). Next, we performed an EGFP‐based retrotransposition assay in mESCs using the L1_RP_‐EGFP transgene 69, 70, 71, 72. This construct has been previously used to track embryonic L1s retrotransposition events in mice *in vivo* 73. The transgene is composed of a L1_RP_ element fused to an EGFP gene (Fig. 5B–C). The EGFP reporter gene is expressed only if the L1_RP_ element completes a full retrotransposition cycle and therefore, assessment of EGFP expression allows the evaluation of the L1 transgene retrotranposition rate (Fig. 5C). The proportion of GFP‐positive cells observed after the L1_RP_‐EGFP transgene transfection is expected to be representative of the number of L1s retrotransposition events, and can be used to compare L1s retrotransposition capacity between mESC lines. As a negative control, we used the L1_JM111_‐EGFP transgene, a mutated version of the L1_RP_‐EGFP transgene, that is unable to retrotranspose (Fig. 5B–C) 73. We transfected both *Dicer* mutants and WT mESCs with the L1_RP_‐EGFP and the L1_JM111_‐EGFP constructs and measured EGFP expression after 3 and 6 weeks by FACS analysis (Fig. 5D–E). No differences between the mESC lines were detected after 3 weeks (Figs 5F and 6). Importantly, after 6 weeks, WT mESCs transfected with the intact construction (pL1_RP_) or with the mutated one (pL1_JM111_) presented similar low levels of GFP‐positive cells, indicating very low retrotransposition activity. However, both *Dicer* mutants transfected with the L1_RP_ vector showed a significant increase (twofold) of GFP‐positive cells compared to their corresponding negative control and to WT mESCs (Figs 5F and 6). We hypothesize that the long period needed is probably due to the high cell mortality observed after transfection and during selection of the *Dicer* mutants. Moreover, the proliferation defect limited the number of cells available for the FACS analysis, thus leading to a possible underestimation of the retrotransposition events in our *Dicer* mutant cells 74, 75. Therefore, we concluded that in the absence of *Dicer*, mESCs accumulate full‐length L1s transcripts and are more permissive to the L1s retrotransposition, demonstrating that *Dicer* is indeed involved in the regulation of L1s retrotransposition in mESCs.

## Conclusion

In this study, we successfully generated and characterized two new independent *Dicer1* knockout (*Dicer*_KO) mESC lines using the CRISPR/Cas9 technology, demonstrating the effectiveness of the paired strategy. This approach allowed us to produce deletions resulting in a complete ablation of the DICER protein function, mimicking the previously generated *Dicer* ^*Cre‐loxP*^ mutants 31, 32, 76. We further demonstrated that *Dicer*_KO mESCs are unable to exit from the pluripotency state and presented a factual reinforcement of the pluripotency network. Therefore, future studies involving the role of *Dicer* in stem cell biology should focus on cellular networks involved in pluripotency exit, an early step of mESCs commitment, rather than in the later stages of the differentiation process 62.

Interestingly, the transcriptome analysis of these mutants revealed a strong accumulation of transcripts from the L1 TE subclasses. We experimentally validated their up‐regulation at mRNA as well as at protein levels. Moreover, we assessed the L1 retrotransposition activity in our *Dicer*_KO and WT mESCs and observed increased retrotransposition events in our mutant cells. However, we did not observe a direct correlation between L1s transcripts abundance and retrotransposition activity in our *Dicer* mutants, as it has been previously reported in human cell lines 77, 78. As retrotransposition events affect only 1% of our mutant cells population (Fig. 5F), it is unlikely that the increased retrotransposition rate is the cause of the exit from pluripotency failure. Nevertheless, the consequential accumulations of L1s transcripts and proteins observed might participate in this inability. For example, the activation of surveillance pathways or quality control mechanisms might prevent cellular differentiation in the presence of increased L1s activity, in order to avert genome instability 79, 80. Importantly, cell survival is intrinsically linked to genome instability 81. Therefore, the increased apoptosis rate observed in our mutants might grant to *Dicer* a potential role in genome integrity maintenance and further support this hypothesis. Interestingly, possible effects of TEs overexpression in mESCs deserve further investigations, as they would imply that a tight monitoring of L1s (and LTR) is essential for normal mammalian development process, due to their essential role in genome integrity. Finally, as miRNAs play a role in the regulation of the transcriptional network controlling pluripotency in mESCs 7, 11, it is therefore possible that DICER is required in the exit from pluripotency process through its role in the biogenesis of miRNAs.

Nevertheless, our work, together with other studies performed in human cultured cells, indicates a role of DICER as a player in L1s regulation 77, 82, 83. How DICER controls L1s still remains unclear and further investigations are needed. Recently, a study performed in human cells indicated that a particular microRNA, miR‐128, was involved in the direct regulation of L1s transcripts 83. Nevertheless, miR‐128 is not expressed in mESCs (data not shown) and therefore cannot explain the regulation of L1s by DICER.

Among the other models proposed, one involves bidirectional transcription of L1 promoters and the potential to generate double‐stranded RNA precursors. These are suitable substrates for DICER resulting in the production of endogenous siRNA, which can trigger repression of the corresponding homologous L1s sequences 1, 84, 85, 86. Indeed, several studies reported the presence of active sense and antisense transcription from human and murine active L1s 77, 87, 88. This model is also supported by the ability of mESCs to produce *Dicer*‐dependent siRNAs 89, and the identification of a population in mESCs of sense and antisense small RNAs mapping to the 5′UTR of active L1_Tf elements 90, 91. To further explore the implication of *Dicer* is this regulation, it would be interesting to monitor L1s expression between the different mutants of the RNAi pathways. These mutants must be generated in the same genetic background to allow their comparison as the TEs composition differs depending on the mouse strains 92.

In conclusion, our results explain the previously observed impaired differentiation process of *Dicer*_KO mESCs and reveal that DICER is essential for the exit from pluripotency of mESCs and the regulation of L1 elements.

## Author contributions

MB and CC conceived study, performed experiments, analyzed data, and wrote the manuscript. DCS and RPN contributed to experiments and data analysis. JY contributed to bioinformatics analysis. All authors read and approved the final manuscript.

## Supporting information


**Table S1.** Primers list.
**Table S2.** Newly generated plasmids.
**Table S3.** Antibodies list.
**Table S4.** Differentially expressed genes in *Dicer_KO* mESCs.

## References

[1] Bernstein E , Caudy AA , Hammond SM and Hannon GJ (2001) Role for a bidentate ribonuclease in the initiation step of RNA interference. Nature 409, 363–366. 11201747 10.1038/35053110

[2] Wilson RC and Doudna JA (2013) Molecular mechanisms of RNA interference. Annu Rev Biophys 42, 217–239. 23654304 10.1146/annurev-biophys-083012-130404 PMC5895182

[3] Foulkes WD , Priest JR and Duchaine TF (2014) DICER1: mutations, microRNAs and mechanisms. Nat Rev Cancer 14, 662–672. 25176334 10.1038/nrc3802

[4] Svoboda P (2014) Renaissance of mammalian endogenous RNAi. FEBS Lett 588, 2550–2556. 24873877 10.1016/j.febslet.2014.05.030

[5] Carthew RW and Sontheimer EJ (2009) Origins and mechanisms of miRNAs and siRNAs. Cell 136, 642–655. 19239886 10.1016/j.cell.2009.01.035 PMC2675692

[6] Wang Y , Baskerville S , Shenoy A , Babiarz JE , Baehner L and Blelloch R (2008) Embryonic stem cell – specific microRNAs regulate the G1‐S transition and promote rapid proliferation. Nat Genet 40, 1478–1483. 18978791 10.1038/ng.250 PMC2630798

[7] Tay Y , Zhang J , Thomson AM , Lim B and Rigoutsos I (2008) MicroRNAs to Nanog, Oct4 and Sox2 coding regions modulate embryonic stem cell differentiation. Nature 455, 1124–1128. 18806776 10.1038/nature07299

[8] Gangaraju VK and Lin H (2009) MicroRNAs: key regulators of stem cells. Nat Rev Mol Cell Biol 10, 116–125. 19165214 10.1038/nrm2621 PMC4118578

[9] Ivey KN and Srivastava D (2010) MicroRNAs as regulators of differentiation and cell fate decisions. Cell Stem Cell 7, 36–41. 20621048 10.1016/j.stem.2010.06.012

[10] Melton C and Blelloch R (2010) Microrna regulation of embryonic stem cell self‐renewal and differentiation. Adv Exp Med Biol 695, 105–117. 21222202 10.1007/978-1-4419-7037-4_8

[11] Gruber AJ , Grandy WA , Balwierz PJ , Dimitrova YA , Pachkov M , Ciaudo C , Van Nimwegen E and Zavolan M (2014) Embryonic stem cell‐specific microRNAs contribute to pluripotency by inhibiting regulators of multiple differentiation pathways. Nucleic Acids Res 42, 9313–9326. 25030899 10.1093/nar/gku544 PMC4132708

[12] Bernstein E , Kim SY , Carmell MA , Murchison EP , Alcorn H , Li MZ , Mills AA , Elledge SJ , Anderson KV and Hannon GJ (2003) Dicer is essential for mouse development. Nat Genet 35, 215–217. 14528307 10.1038/ng1253

[13] Yang WJ , Yang DD , Na S , Sandusky GE , Zhang Q and Zhao G (2005) Dicer is required for embryonic angiogenesis during mouse development. J Biol Chem 280, 9330–9335. 15613470 10.1074/jbc.M413394200

[14] Svoboda P , Stein P , Anger M , Bernstein E , Hannon GJ and Schultz RM (2004) RNAi and expression of retrotransposons MuERV‐L and IAP in preimplantation mouse embryos. Dev Biol 269, 276–285. 15081373 10.1016/j.ydbio.2004.01.028

[15] Obbard DJ , Gordon KHJ , Buck AH and Jiggins FM (2009) The evolution of RNAi as a defence against viruses and transposable elements. Philos Trans R Soc Lond B Biol Sci 364, 99–115. 18926973 10.1098/rstb.2008.0168 PMC2592633

[16] Maillard PV , Ciaudo C , Marchais A , Li Y , Jay F , Ding SW and Voinnet O (2013) Antiviral RNA interference in mammalian cells. Science 342, 235–238. 24115438 10.1126/science.1241930 PMC3853215

[17] Keller G (2005) Embryonic stem cell differentiation : emergence of a new era in biology and medicine. Genes Dev 19, 1129–1155. 15905405 10.1101/gad.1303605

[18] Howlett SK and Reik W (1991) Methylation levels of maternal and paternal genomes during preimplantation development. Development 113, 119–127. 1764989 10.1242/dev.113.1.119

[19] Ohnishi Y , Totoki Y , Toyoda A , Watanabe T , Yamamoto Y , Tokunaga K , Sakaki Y , Sasaki H and Hohjoh H (2010) Small RNA class transition from siRNA/piRNA to miRNA during pre‐implantation mouse development. Nucleic Acids Res 38, 5141–5151. 20385573 10.1093/nar/gkq229 PMC2926599

[20] Finnegan DJ (1989) Eukaryotic transposable elements and genome evolution. Trends Genet 5, 103–107. 2543105 10.1016/0168-9525(89)90039-5

[21] Lander ES , Linton LM , Birren B , Nusbaum C , Zody MC , Baldwin J , Devon K , Dewar K , Doyle M , FitzHugh W *et al* (2001) International Human Genome Sequencing, Initial sequencing and analysis of the human genome. Nature 409, 860–921. 11237011 10.1038/35057062

[22] Waterston RH , Lindblad‐Toh K , Birney E , Rogers J , Abril JF , Agarwal P , Agarwala R , Ainscough R , Alexandersson M , An P *et al* (2002) Mouse Genome Sequencing, Initial sequencing and comparative analysis of the mouse genome. Nature 420, 520–562. 12466850 10.1038/nature01262

[23] Wicker T , Sabot F , Hua‐Van A , Bennetzen JL , Capy P , Chalhoub B , Flavell A , Leroy P , Morgante M , Panaud O *et al* (2007) A unified classification system for eukaryotic transposable elements. Nat Rev Genet 8, 973–982. 17984973 10.1038/nrg2165

[24] Bodak M , Yu J and Ciaudo C (2014) Regulation of LINE‐1 in mammals. Biomol Concepts 5, 409–428. 25367621 10.1515/bmc-2014-0018

[25] Ostertag EM and Kazazian HHJ (2001) Biology of mammalian L1. Annu Rev Genet 35, 501–538. 11700292 10.1146/annurev.genet.35.102401.091032

[26] Naas TP , Deberardinis RJ , Moran JV , Ostertag EM , Kingsmore SF , Seldin MF , Hayashizaki Y , Martin SL and Kazazian HH (1998) An actively retrotransposing, novel subfamily of mouse L1 elements. EMBO J 17, 590–597. 9430649 10.1093/emboj/17.2.590 PMC1170408

[27] Goodier JL , Ostertag EM , Du K and Kazazian HH Jr (2001) A novel active L1 retrotransposon subfamily in the mouse. Genome Res 11, 1677–1685. 11591644 10.1101/gr.198301 PMC311137

[28] Brouha B , Schustak J , Badge RM , Lutz‐Prigge S , Farley AH , Moran JV and Kazazian HH (2003) Hot L1s account for the bulk of retrotransposition in the human population. Proc Natl Acad Sci USA 100, 5280–5285. 12682288 10.1073/pnas.0831042100 PMC154336

[29] Beck CR , Garcia‐Perez JL , Badge RM and Moran JV (2011) LINE‐1 elements in structural variation and disease. Annu Rev Genomics Hum Genet 12, 187–215. 21801021 10.1146/annurev-genom-082509-141802 PMC4124830

[30] Kazazian HH (2004) Mobile elements: drivers of genome evolution. Science 303, 1626–1632. 15016989 10.1126/science.1089670

[31] Kanellopoulou C , Muljo SA , Kung AL , Ganesan S , Drapkin R , Jenuwein T , Livingston DM and Rajewsky K (2005) Dicer‐deficient mouse embryonic stem cells are defective in differentiation and centromeric silencing. Genes Dev 19, 489–501. 15713842 10.1101/gad.1248505 PMC548949

[32] Murchison EP , Partridge JF , Tam OH , Cheloufi S and Hannon GJ (2005) Characterization of Dicer‐deficient murine embryonic stem cells. Proc Natl Acad Sci USA 102, 12135–12140. 16099834 10.1073/pnas.0505479102 PMC1185572

[33] Bodak M and Ciaudo C (2016) Monitoring long interspersed nuclear element 1 expression during mouse embryonic stem cell differentiation. Methods Mol Biol 1400, 237–259. 26895058 10.1007/978-1-4939-3372-3_16

[34] Wettstein R , Bodak M and Ciaudo C (2015) Generation of a knockout mouse embryonic stem cell line using a paired CRISPR/Cas9 Genome engineering tool. Methods Mol Biol 1341, 321–343. 10.1007/7651_2015_213 25762293

[35] Heigwer F , Kerr G and Boutros M (2014) E‐CRISP: fast CRISPR target site identification. Nat Methods 11, 122–123. 24481216 10.1038/nmeth.2812

[36] Koike‐Yusa H , Li Y , Tan E‐P , Velasco‐Herrera MDC and Yusa K (2013) Genome‐wide recessive genetic screening in mammalian cells with a lentiviral CRISPR‐guide RNA library. Nat Biotechnol 32, 267–273. 24535568 10.1038/nbt.2800

[37] Cong L , Ran FA , Cox D , Lin S , Barretto R , Hsu PD , Wu X , Jiang W and Marraffini LA (2013) Multiplex genome engineering using CRISPR/Cas systems. Science 339, 819–823. 23287718 10.1126/science.1231143 PMC3795411

[38] Jay F and Ciaudo C (2013) An RNA tool kit to study the status of mouse ES cells: sex determination and stemness. Methods 63, 85–92. 23473779 10.1016/j.ymeth.2013.02.016

[39] Kuramochi‐Miyagawa S , Watanabe T , Gotoh K , Totoki Y , Toyoda A , Ikawa M , Asada N , Kojima K , Yamaguchi Y , Ijiri TW *et al* (2008) DNA methylation of retrotransposon genes is regulated by Piwi family members MILI and MIWI2 in murine fetal testes. Genes Dev 22, 908–917. 18381894 10.1101/gad.1640708 PMC2279202

[40] Bolger AM , Lohse M and Usadel B (2014) Trimmomatic: a flexible trimmer for Illumina sequence data. Bioinformatics 30, 2114–2120. 24695404 10.1093/bioinformatics/btu170 PMC4103590

[41] Dobin A , Davis CA , Schlesinger F , Drenkow J , Zaleski C , Jha S , Batut P , Chaisson M and Gingeras TR (2013) STAR: ultrafast universal RNA‐seq aligner. Bioinformatics 29, 15–21. 23104886 10.1093/bioinformatics/bts635 PMC3530905

[42] Liao Y , Smyth GK and Shi W (2014) FeatureCounts: an efficient general purpose program for assigning sequence reads to genomic features. Bioinformatics 30, 923–930. 24227677 10.1093/bioinformatics/btt656

[43] Ginestet C (2011) Ggplot2: elegant graphics for data analysis. J R Stat Soc 174, 244–245.

[44] Jin Y , Tam OH , Paniagua E and Hammell M (2015) TEtranscripts: a package for including transposable elements in differential expression analysis of RNA‐seq datasets. Bioinformatics 31, 3593–3599. 26206304 10.1093/bioinformatics/btv422 PMC4757950

[45] Robinson MD , McCarthy DJ and Smyth GK (2009) edgeR: A Bioconductor package for differential expression analysis of digital gene expression data. Bioinformatics 26, 139–140. 19910308 10.1093/bioinformatics/btp616 PMC2796818

[46] Cirera‐Salinas D , Pauta M , Allen RM , Salerno AG , Ramírez CM , Chamorro‐Jorganes A , Wanschel AC , Lasunción MA , Morales‐Ruiz M , Suárez Y *et al* (2012) Mir‐33 regulates cell proliferation and cell cycle progression. Cell Cycle 11, 922–933. 22333591 10.4161/cc.11.5.19421 PMC3323796

[47] Riccardi C and Nicoletti I (2006) Analysis of apoptosis by propidium iodide staining and flow cytometry. Nat Protoc 1, 1458–1461. 17406435 10.1038/nprot.2006.238

[48] Abràmoff MD , Hospitals I , Magalhães PJ and Abràmoff M (2004) Image processing with ImageJ. Biophotonics Int 11, 36–42.

[49] Jinek M , East A , Cheng A , Lin S , Ma E and Doudna J (2013) RNA‐programmed genome editing in human cells. Elife 2013, 1–9. 10.7554/eLife.00471 PMC3557905 23386978

[50] Hsu PD , Lander ES , Zhang F , Sciences C and Biology C (2014) Development and applications of CRIPR‐Cas9 for genome engineering. Cell 157, 1262–1278. 24906146 10.1016/j.cell.2014.05.010 PMC4343198

[51] Canver MC , Bauer DE , Dass A , Yien YY , Chung J , Masuda T , Maeda T , Paw BH and Orkin SH (2014) Characterization of genomic deletion efficiency mediated by clustered regularly interspaced palindromic repeats (CRISPR)/cas9 nuclease system in mammalian cells. J Biol Chem 289, 21312–21324. 24907273 10.1074/jbc.M114.564625 PMC4118095

[52] Houbaviy HB , Murray MF and Sharp PA (2003) Embryonic stem cell‐specific MicroRNAs. Dev Cell 5, 351–358. 12919684 10.1016/s1534-5807(03)00227-2

[53] Landgraf P , Rusu M , Sheridan R , Sewer A , Iovino N , Aravin A , Pfeffer S , Rice A , Kamphorst AO , Lin C *et al* (2007) A mammalian microRNA expression atals based on small RNA library sequencing. Cell 129, 1401–1414. 17604727 10.1016/j.cell.2007.04.040 PMC2681231

[54] Martinez NJ and Gregory RI (2013) Argonaute2 expression is post‐transcriptionally coupled to microRNA abundance. RNA 19, 605–612. 23485552 10.1261/rna.036434.112 PMC3677276

[55] Bueno MJ and Malumbres M (1812) MicroRNAs and the cell cycle. Biochim Biophys Acta 2011, 592–601. 10.1016/j.bbadis.2011.02.002 21315819

[56] Su Z , Yang Z , Xu Y , Chen Y and Yu Q (2015) MicroRNAs in apoptosis, autophagy and necroptosis. Oncotarget 6, 8474–8490. 25893379 10.18632/oncotarget.3523 PMC4496162

[57] Young RA (2011) Control of the embryonic stem cell state. Cell 144, 940–954. 21414485 10.1016/j.cell.2011.01.032 PMC3099475

[58] Yeo J‐C and Ng H‐H (2013) The transcriptional regulation of pluripotency. Cell Res 23, 20–32. 23229513 10.1038/cr.2012.172 PMC3541660

[59] Kalkan T and Smith A (2014) Mapping the route from naive pluripotency to lineage specification. Philos Trans R Soc Lond B Biol Sci 369, 20130540. 25349449 10.1098/rstb.2013.0540 PMC4216463

[60] Ying Q‐L , Stavridis M , Griffiths D , Li M and Smith A (2003) Conversion of embryonic stem cells into neuroectodermal precursors in adherent monoculture. Nat Biotechnol 21, 183–186. 12524553 10.1038/nbt780

[61] Ying QL , Wray J , Nichols J , Batlle‐Morera L , Doble B , Woodgett J , Cohen P and Smith A (2008) The ground state of embryonic stem cell self‐renewal. Nature 453, 519–523. 18497825 10.1038/nature06968 PMC5328678

[62] Betschinger J , Nichols J , Dietmann S , Corrin PD , Paddison PJ and Smith A (2013) Exit from pluripotency is gated by intracellular redistribution of the bHLH transcription factor Tfe3. Cell 153, 335–347. 23582324 10.1016/j.cell.2013.03.012 PMC3661979

[63] Annere C , Tamm C and Galito SP (2013) A comparative study of protocols for mouse embryonic stem cell culturing. PLoS ONE 8, 1–10. 10.1371/journal.pone.0081156 PMC3858223 24339907

[64] Wray J , Kalkan T , Gomez‐Lopez S , Eckardt D and Cook A (2012) Inhibition of glycogen synthase kinase‐3 alleviates Tcf3 repression of the pluripotency network and increases embryonic stem cell resistance to differentiation. Nat Cell Biol 13, 838–845. 10.1038/ncb2267 PMC3160487 21685889

[65] Marks H , Kalkan T , Menafra R , Denissov S , Jones K , Hofemeister H , Nichols J , Kranz A , Stewart AF , Smith A *et al* (2012) The transcriptional and epigenomic foundations of ground state pluripotency. Cell 149, 590–604. 22541430 10.1016/j.cell.2012.03.026 PMC3398752

[66] Chambers I , Silva J , Colby D , Nichols J , Nijmeijer B , Robertson M , Vrana J , Jones K , Grotewold L and Smith A (2007) Nanog safeguards pluripotency and mediates germline development. Nature 450, 3–8. 10.1038/nature06403 18097409

[67] Hayashi K , Chuva SM , Lopes DS , Tang F and Surani MA (2008) Article dynamic equilibrium and heterogeneity of mouse pluripotent stem cells with distinct functional and epigenetic states. Cell Stem Cell 3, 391–401. 18940731 10.1016/j.stem.2008.07.027 PMC3847852

[68] Cui L , Johkura K , Yue F , Ogiwara N , Okouchi Y and Asanuma K (2004) Spatial distribution and initial changes of SSEA‐1 and other cell adhesion‐related molecules on mouse embryonic stem cells before and during differentiation the journal of histochemistry & cytochemistry. J Histochem Cytochem 52, 1447–1457. 15505339 10.1369/jhc.3A6241.2004 PMC3957812

[69] Heidmann O and Heidmann T (1991) Retrotransposition of a mouse IAP sequence tagged with an indicator gene. Cell 64, 159–170. 1846087 10.1016/0092-8674(91)90217-m

[70] Moran J , Holmes S and Naas T (1996) High frequency retrotransposition in cultured mammalian cells. Cell 87, 917–927. 8945518 10.1016/s0092-8674(00)81998-4

[71] Ostertag EM , Prak ET , DeBerardinis RJ , Moran JV and Kazazian HH Jr (2000) Determination of L1 retrotransposition kinetics in cultured cells. Nucleic Acids Res 28, 1418–1423. 10684937 10.1093/nar/28.6.1418 PMC111040

[72] Ostertag EM , DeBerardinis RJ , Goodier JL , Zhang Y , Yang N , Gerton GL and Kazazian HH (2002) A mouse model of human L1 retrotransposition. Nat Genet 32, 655–660. 12415270 10.1038/ng1022

[73] Prak ETL , Dodson AW , Farkash EA and Kazazian HH (2003) Tracking an embryonic L1 retrotransposition event. Proc Natl Acad Sci USA 100, 1832–1837. 12569170 10.1073/pnas.0337627100 PMC149919

[74] Shi X , Seluanov A and Gorbunova V (2007) Cell divisions are required for L1 retrotransposition. Mol Cell Biol 27, 1264–1270. 17145770 10.1128/MCB.01888-06 PMC1800731

[75] Xie Y , Mates L , Ivics Z , Izsvák Z , Martin SL and An W (2013) Cell division promotes efficient retrotransposition in a stable L1 reporter cell line. Mob DNA 4, 1–10. 23497436 10.1186/1759-8753-4-10 PMC3607998

[76] Macrae IJ , Zhou K , Li F , Repic A , Brooks AN , Cande WZ , Adams PD and Doudna JA (2006) Structural basis for double‐stranded RNA processing by Dicer. Science 195, 195–198. 10.1126/science.1121638 16410517

[77] Yang N and Kazazian HH (2006) L1 retrotransposition is suppressed by endogenously encoded small interfering RNAs in human cultured cells. Nat Struct Mol Biol 13, 763–771. 16936727 10.1038/nsmb1141

[78] Yang N , Zhang L , Zhang Y and Kazazian HH (2003) An important role for RUNX3 in human L1 transcription and retrotransposition. Nucleic Acids Res 31, 4929–4940. 12907736 10.1093/nar/gkg663 PMC169909

[79] Malki S , van der Heijden GW , O'Donnell KA , Martin SL and Bortvin A (2014) A role for retrotransposon LINE‐1 in fetal oocyte attrition in mice. Dev Cell 29, 521–533. 24882376 10.1016/j.devcel.2014.04.027 PMC4056315

[80] Taylor MS , Lacava J , Mita P , Molloy KR , Ran C , Huang L , Li D , Adney EM , Jiang H , Burns KH *et al* (2013) Affinity proteomics reveals human host factors implicated in discrete stages of LINE‐1 retrotransposition. Cell 155, 1034–1048. 24267889 10.1016/j.cell.2013.10.021 PMC3904357

[81] Zhivotovsky B and Kroemer G (2004) Apoptosis and genomic instability. Nat Rev Mol Cell Biol 5, 752–762. 15340382 10.1038/nrm1443

[82] Soifer HS , Zaragoza A , Peyvan M , Behlke MA and Rossi JJ (2005) A potential role for RNA interference in controlling the activity of the human LINE‐1 retrotransposon. Nucleic Acids Res 33, 846–856. 15701756 10.1093/nar/gki223 PMC549394

[83] Hamdorf M , Idica A , Zisoulis DG , Gamelin L , Martin C , Sanders KJ and Pedersen IM (2015) miR‐128 represses L1 retrotransposition by binding directly to L1 RNA. Nat Struct Mol Biol 22, 824–831. 26367248 10.1038/nsmb.3090

[84] Martinez J , Patkaniowska A , Urlaub H , Lührmann R and Tuschl T (2002) Single‐stranded antisense siRNAs guide target RNA cleavage in RNAi. Cell 110, 563–574. 12230974 10.1016/s0092-8674(02)00908-x

[85] Bayne EH and Allshire RC (2005) RNA‐directed transcriptional gene silencing in mammals. Trends Genet 21, 370–373. 15908035 10.1016/j.tig.2005.05.007

[86] Kim D‐H , Behlke MA , Rose SD , Chang M‐S , Choi S and Rossi JJ (2005) Synthetic dsRNA Dicer substrates enhance RNAi potency and efficacy. Nat Biotechnol 23, 222–226. 15619617 10.1038/nbt1051

[87] Speek M (2001) Antisense promoter of human L1 retrotransposon drives transcription of adjacent cellular genes. Mol Cell Biol 21, 1973–1985. 11238933 10.1128/MCB.21.6.1973-1985.2001 PMC86790

[88] Li J , Kannan M , Trivett AL , Liao H , Wu X , Akagi K and Symer DE (2014) An antisense promoter in mouse L1 retrotransposon open reading frame‐1 initiates expression of diverse fusion transcripts and limits retrotransposition. Nucleic Acids Res 42, 4546–4562. 24493738 10.1093/nar/gku091 PMC3985663

[89] Babiarz JE , Ruby JG , Wang Y , Bartel DP and Blelloch R (2008) Mouse ES cells express endogenous shRNAs, siRNAs, and other microprocessor‐independent, dicer‐dependent small RNAs. Genes Dev 22, 2773–2785. 18923076 10.1101/gad.1705308 PMC2569885

[90] Chow JC , Ciaudo C , Fazzari MJ , Mise N , Servant N , Glass JL , Attreed M , Avner P , Wutz A , Barillot E *et al* (2010) LINE‐1 activity in facultative heterochromatin formation during X chromosome inactivation. Cell 141, 956–969. 20550932 10.1016/j.cell.2010.04.042

[91] Toedling J , Servant N , Ciaudo C , Farinelli L , Voinnet O , Heard E and Barillot E (2012) Deep‐sequencing protocols influence the results obtained in small‐RNA sequencing. PLoS ONE 7, e32724. 22384282 10.1371/journal.pone.0032724 PMC3287988

[92] Nellåker C , Keane TM , Yalcin B , Wong K , Agam A , Belgard TG , Flint J , Adams DJ , Frankel WN and Ponting CP (2012) The genomic landscape shaped by selection on transposable elements across 18 mouse strains. Genome Biol 13, 159. 22703977 10.1186/gb-2012-13-6-r45 PMC3446317

[93] Kamburov A , Pentchev K , Galicka H , Wierling C , Lehrach H and Herwig R (2011) ConsensusPathDB: toward a more complete picture of cell biology. Nucleic Acids Res 39, 712–717. 21071422 10.1093/nar/gkq1156 PMC3013724

[94] Kamburov A , Stelzl U , Lehrach H , Herwig R (2013) The Consensus PathDB and interaction database: 2013 Update. Nucleic Acids Res 41, 793–800. 10.1093/nar/gks1055 PMC3531102 23143270

[95] Loeb DD , Padgett RW , Hardies SC , Shehee WRON , Comer MB , Edgell MH and Hutchison CA III (1986) The sequence of a large LlMd element reveals a tandemly repeated 5′ end and several features found in retrotransposons. Mol Cell Biol 6, 168–182. 3023821 10.1128/mcb.6.1.168-182.1986 PMC367496

[96] Deberardinis RJ and Kazazian HH (1999) Analysis of the promoter from an expanding mouse retrotransposon subfamily. Genomics 323, 317–323. 10.1006/geno.1998.5729 10087199

